# Supplementary material for: Active site specificity profiling datasets of matrix metalloproteinases (MMPs) 1, 2, 3, 7, 8, 9, 12, 13 and 14
Source: Data Brief. 2016 Feb 22;7:299–310. doi: 10.1016/j.dib.2016.02.036 (PMC4777984; doi:10.1016/j.dib.2016.02.036)
Supplement: Supplementary file 10 — Supplementary material [file mmc10.zip › WebPICS_hMMP1_G_1%/P1prime.html]

 

PICS results


|  |  |
| --- | --- |
| **P1prime\_C**  8 in 126 sites   6.3 %    effects > 10 perc. pnts.  (vice-versa in brackets)  P3\_T: 28.8 (21.0)   P2\_A: 15.5 (10.4)   P2\_Y: 15.5 (10.4)   P1\_S: 34.9 (14.8)   P2prime\_T: 29.6 (23.7)   P3prime\_T: 16.3 (11.9) |  |
| **P1prime\_I**  25 in 126 sites   19.8 %    effects > 10 perc. pnts.  (vice-versa in brackets)  P3\_P: 12.2 (12.2)   P2\_Y: 14.5 (30.2)   P1\_S: -11.1 (-14.5) |  |
| **P1prime\_L**  62 in 126 sites   49.2 %    effects > 10 perc. pnts.  (vice-versa in brackets)  P3\_P: -10.1 (-25.2) |  |
| **P1prime\_M**  6 in 126 sites   4.8 %    effects > 10 perc. pnts.  (vice-versa in brackets)  P3prime\_T: 41.3 (22.5) |  |
